# Plant Neighbour Identity Matters to Belowground Interactions under Controlled Conditions

**DOI:** 10.1371/journal.pone.0027791

**Published:** 2011-11-17

**Authors:** Cristina Armas, Francisco Ignacio Pugnaire

**Affiliations:** Grupo de Ecología Funcional, Departmento de Ecología Funcional y Evolutiva, Estación Experimental de Zonas Áridas, Consejo Superior de Investigaciones Científicas, Almería, Spain; University Copenhagen, Denmark

## Abstract

**Background:**

Root competition is an almost ubiquitous feature of plant communities with profound effects on their structure and composition. Far beyond the traditional view that plants interact mainly through resource depletion (exploitation competition), roots are known to be able to interact with their environment using a large variety of mechanisms that may inhibit or enhance access of other roots to the resource or affect plant growth (contest interactions). However, an extensive analysis on how these contest root interactions may affect species interaction abilities is almost lacking.

**Methodology/Principal Findings:**

In a common garden experiment with ten perennial plant species we forced pairs of plants of the same or different species to overlap their roots and analyzed how belowground contest interactions affected plant performance, biomass allocation patterns, and competitive abilities under abundant resource supply. Our results showed that net interaction outcome ranged from negative to positive, affecting total plant mass and allocation patterns. A species could be a strong competitor against one species, weaker against another one, and even facilitator to a third species. This leads to sets of species where competitive hierarchies may be clear but also to groups where such rankings are not, suggesting that intransitive root interactions may be crucial for species coexistence.

**Conclusions/Significance:**

The outcome of belowground contest interactions is strongly dependent on neighbours' identity. In natural plant communities this conditional outcome may hypothetically help species to interact in non-hierarchical and intransitive networks, which in turn might promote coexistence.

## Introduction

A central theme in community ecology is the role of plant interactions in determining species coexistence and performance in communities [1], but most research on this topic usually focussed on competition. While intraspecific competition tends to be intense and density-dependent [2], [3], interspecific competition can be intense, weak, or negligible as the different species differ in morphology and requirements [4], [5]. However, evidence suggests that intraspecific competition is not necessarily more intense than interspecific competition [4], [6]. For example, there is evidence that intraspecific competition may be decreased by reducing overlap between roots of conspecific neighbours [6]–[9]. Overall, complex combinations of competitive and facilitative interactions do occur simultaneously [10] and the net balance of interspecific interactions will depend on the relative importance of positive and negative influences exerted by each species on the other [11].

Belowground plant interactions have been mostly linked to nutrients and water uptake, in contrast to aboveground interactions, which are commonly linked to carbon and energy acquisition [2]. Root interactions usually alter the availability of belowground resources, positively or negatively affecting neighbours. Resource depletion has traditionally been regarded as the main mechanism determining belowground plant interactions (i.e. exploitation competition *sensu* Schenk [6]). But belowground facilitation also exists, and usually imply an increase in resources, e.g. water or nutrients *via* hydraulic lift [12], [13], carbon and water exchanges through mycorrhizal networks [14], [15], or direct transfer of nutrients between plants [16], [17].

There is, however, increasing awareness that roots do not interact solely through depletion/enhancement of soil resources, but they may also interact by mechanisms that inhibit or enhance access of other roots to soil resources or affect plant growth (i.e., contest competition *sensu* Schenk [6]). Roots may directly interfere or inhibit growth of neighbouring roots [18], avoid each other [8], proliferate in the presence of other roots [19], [20] or behave differently when encountering ‘self’ vs. ‘non-self’ roots [21]. Through root exudates, plants may regulate the soil microbial community in their vicinity, cope with herbivores, encourage beneficial symbioses, and change chemical properties of soils [22]–[24]. All these signalling mechanisms and chemical interactions direct and indirectly affect the net outcome of plant interactions [25]–[27], with profound impacts on communities and ecosystems[6]. The diverse nature of the mechanisms behind root contest interactions suggests they should be highly species-specific. However, the general effects of these belowground contest interactions on plant performance, biomass allocation and how they may affect species potential interaction abilities have seldom been tested.

In a common garden experiment we forced root contact in pairs of saplings of 10 perennial species by overlapping their rhizospheres (Tables 1 and 2). Resource availability (water, nutrients, light, and soil volume) was the same in all cases irrespective of whether there was one plant growing alone or with a neighbour. We tried to minimize resource exploitation by minimizing competition for light and belowground resources, expecting to be able to identify plant responses attributable to root contest interactions (either competition or facilitation). We expected that 1) the outcome of root contest interactions will be species-specific; although some species may be better competitors than others, for most species the outcome of root interactions will depend on neighbours' identity; and 2) may differentially affect biomass allocation to above and belowground plant parts. In addition, conspecific plants are, by definition, similar in demands and abilities regarding resource acquisition and therefore we expected that 3) competitive effects will predominate between conspecific individuals, which will grow smaller than isolated plants and will allocate more biomass to roots than isolated plants in response to contest root competition.

**Table 1:** Species used in this study, identifying symbol in figures, and number of plants used for each species. *Genista spartioides* was only used as a neighbour species but not as target.

Species	Life form	Symbol	# Plants
*Anthyllis cytisoides* L.	Small shrub	Ac	29
*Limonium insigne* (Cosson) O. Kuntze	Small shrub	Li	56
*Lygeum spartum* L.	Tussock grass	Ls	71
*Olea europaea* L.	Small tree	Oe	37
*Pinus halepensis* Mill.	Tree	Ph	67
*Quercus coccifera* L.	Small tree	Qc	54
*Quercus suber* L.	Tree	Qs	31
*Retama sphaerocarpa* L.	Big shrub	Rs	38
*Stipa tenacissima* L.	Tussock grass	St	78
*Genista spartioides* Spach	Shrub	Gs	17

**Table 2:** Experimental design.

Interaction	N	# Pots	Interaction	N	# Pots
***A. cytisoides***	**6**	**6**	***Q. suber***	**13**	**13**
Ac-Ac	8	4	Qs-Qs	8	4
Ac-Gs	4	4	Qs-Oe	5	5
Ac-Rs	8	8	Qs-Qc	5	
Ac-St	3	3	***R. sphaerocarpa***	**9**	**9**
***L. insigne***	**14**	**14**	Rs-Rs	6	3
Li-Li	22	11	Rs-Ac	8	
Li-Ls	13	13	Rs-Gs	6	6
Li-St	7	7	Rs-St	9	9
***L spartum***	**15**	**15**	***S. tenacissima***	**13**	**13**
Ls-Ls	30	15	St-St	16	8
Ls-Li	13		St-Ac	3	
Ls-St	13	13	St-Gs	7	7
***P. halepensis***	**12**	**12**	St-Li	7	
Ph-Ph	26	13	St-Ls	13	
Ph-Oe	13	13	St-Ph	10	
Ph-Qc	6	6	St-Rs	9	
Ph-St	10	10	***O. europaea***	**12**	**12**
***Q. coccifera***	**14**	**14**	Oe-Ph	13	
Qc-Qc	22	11	Oe-Qc	7	
Qc-Oe	7	7	Oe-Qs	5	
Qc-Ph	6				
Qc-Qs	5	5	**# (Plants or pots)**	**461**	**293**

Bold cells indicate the control treatment (plant growing isolated in the pot). N indicates the number of replicates (plants) for the first species that appears in the first column, and from which we measured the effect of the associated plant species in the pot (indicated by the second species in the first column). Total #Plants does not coincide with the number of plants used in the experiment (Table 1) as here we did not include *Genista spartioides* individuals. # Pots: number of experimental units. Blank cells correspond to pots already counted. Legend of species symbols is as in Table 1.

## Materials and Methods

### Experimental design and species selection

We selected 10 species including trees, shrubs, and perennial grasses (Table 1) common in Mediterranean and semiarid plant communities in SE Spain. Seeds were collected from the field and germinated in a nursery in Rodalquilar (Almería, Spain, 37°N, 02°W, 50 m elevation). Climate is Mediterranean semiarid with an annual mean of 150 mm of precipitation, 18°C of temperature and mean total radiation 34–62 molm^−2^d^−1^.

After germination, seedlings were planted in individual pots where they grew isolated for two years, being watered to field capacity every day. We then carefully washed away the potting mix and each sapling was planted into 300 cm^3^ containers filled with sphagnum peat. We tried to maximize root contact between individuals, and placed roots in intimate contact with each-other while placing canopies as far apart as possible. Given the small size of plants and the high irradiance in the nursery area (34–62 molm^−2^d^−1^) we assumed that aboveground interactions -competition for light– was minimal compared to belowground interactions (see results).

We established three treatments, intra- and interspecific interactions, in which plants were paired either with a conspecific or an individual of a different species, and no-interaction (controls), in which individual plants grew isolated (Table 2). There were controls for all species except for *Genista spartioides*. Overall, there were 8 cases of intra-specific interactions, including all species but *Genista* and *Olea europaea*, and 14 inter-specific interactions (Table 2).

We only paired species that coexisted in natural plant communities. The initial number of replicates was 15. Some individuals died shortly after transplant most likely because of root manipulations. We followed a conservative approach and excluded from analysis all pots where one of the plants was dead (Table 2).

After transplant, plants grew for one year in the nursery under the same (optimal) growth conditions, regularly supplied with nutrients and water (nutrients were applied as a slow-release commercial fertilizer and soils were watered to field capacity once a day). At the end of the experiment, plants were harvested and plant material was separated into leaves, shoots, and roots. Samples were oven-dried at 70°C for 72 hours, and weighed.

### Variables measured

On each individual we measured total dry mass, the root-to-shoot biomass ratio (*R:S*), and the intensity of the interaction of a species on neighbour species as a function of biomass. We used a relative interaction index, RII [28] as a metric of interaction intensity, which is defined as
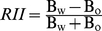
(1)where B_w_ is the mass of an individual growing with another plant and B_o_ is the mean value of control plants of the same species. RII has defined limits [−1, +1], being negative when competition prevails, positive for prevalence of facilitation and 0 when the net balance of the interaction is neutral.

### Statistical analysis

Differences were tested using ANOVA at a significance level of 0.05. These ANOVA tests did not follow a complete factorial design as only the species allowed to interact where those coexisting in natural communities. Previous to ANOVA, we tested whether source habitat may potentially modulate the results by ANCOVA, and found that it did not, neither for biomass nor interaction intensity (results not shown). We thus proceeded with one-way ANOVA tests. Homogeneity of variances was checked using Levene's test. When variables were heteroscadistic we applied the Brown-Forsythe and Welch statistics, as they are more robust than the *F* of Fisher for such data [29]. When variables were heteroscadistic and there was a significant correlation between variance and mean values we applied the alternative Kruskal-Wallis non-parametric test. Post-hoc differences were explored with Hochberg's GT2 test, recommended for unbalanced designs. If data were heteroscadistic, post-hoc differences were explored with Tamhane's T2 test. Biomass and R:S ratios were log-transformed to normalize their distribution.

Differences in response to neighbour identity and the effect of one target species on neighbour species (RII) were tested with one-way ANOVA for each species separately. Isolated plants were used as control. Positive and negative values of RII were considered to represent significant net positive (i.e. facilitation) and negative (i.e. competition) effects only when they statistically differ from zero (i.e. zero denotes no interaction or neutral effect of the interaction) and only when the biomass of plants of a species growing with a neighbor species significantly differ from their control treatment, i.e. isolated plants.

All analyses were performed with the SPSS v.17.0 (SPSS Inc., Chicago, IL, USA). Data presented throughout the text are mean values ±1 SE.

## Results

### Plant growth and biomass allocation patterns

**Figure 1:**
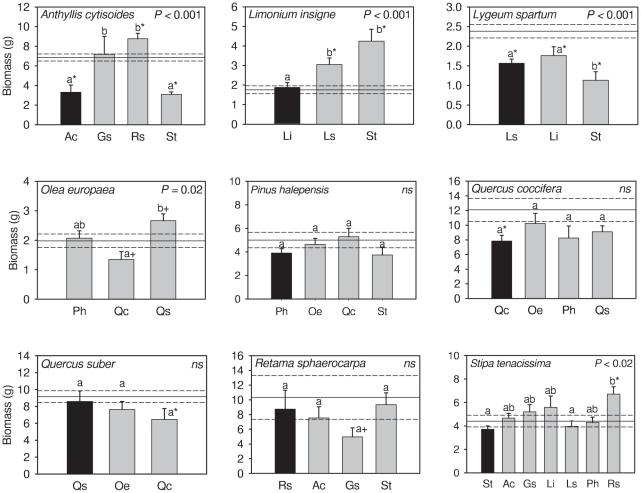
Total mass of individuals of target species (name inside the panel) growing in association to individuals of the same (solid column) or different species (grey column). *P* is the significance from ANOVA (*ns*: non-significant differences). The continuous horizontal line shows the mean mass of control plants and dashed lines represent ±1 SE. Bars with different letters are significantly different at *P*<0.05. * (*P*<0.05) and ^+^ (*P*<0.07) show differences with control plants. Legend of species is as in Table 1.

Final mass of isolated individuals differed between species (*F*_8,107_ = 34.46, *P*<0.001) and biomass was overall affected by the presence of neighbours (4.60 ± 0.18 *vs*. 5.78 ± 0.49 g for plants with neighbours and controls, respectively; *F*_1,456_ = 4.78, *P*<0.05). Six out of the 9 species tested were affected by growing with a neighbour (Fig 1), and in half of the 22 pairs analyzed at least one of the species had different mass than their controls: i.e., there was a net competitive or facilitative effect. In 3 more cases differences were also marginally significant (*P*<0.07, Fig 1).

Contest competition seemed to be the dominant outcome in intraspecific interactions, but it was only significant for 3 out of 8 cases; in *Anthyllis cytisoides*, *Lygeum spartum* and *Quercus coccifera*, where individuals growing with a conspecific were much smaller than when isolated. On the contrary, the effects of interspecific interactions were more variable; plants growing with individuals of other species had either smaller (4 cases out of 27, plus 2 more cases with marginally significant interactions), greater (4 cases, plus 1 marginally significant) or similar (the remaining 16 cases) mass than control individuals (Fig 1).

Overall, biomass tended to be smaller for individuals growing with a conspecific than for individuals growing with other species (*F*_1,348_ = 3.87, *P* = 0.05). The only exception was for *Lygeum* accompanied by *Stipa tenacissima* (Fig 1).

Changes in biomass due to the presence of neighbours were more evident in grasses (*Lygeum* and *Stipa*) and small shrubs (*Anthyllis* and *Limonium insigne*) than in bigger shrubs or trees. It is worth noticing that plants accompanied by a legume tended to have greater mass, except if both were legumes. This facilitative effect was not restricted to legumes. Grasses (*Lygeum* and *Stipa*) had a positive effect on *Limonium*, and *Quercus suber* also tended to improve *Olea* growth (*P*<0.07, Fig 1).

Biomass allocation patterns (*R:S*) were variable and species-specific (*F*_8,107_ = 42.57, *P<*0.001), being affected by neighbour identity (*F*_10,456_ = 6.18, *P<*0.001). Positive and negative interactions –based on differences in total biomass compared to controls- did affect plant allocation patterns in disparate ways. Competition did not change *R:S* ratio compared to isolated plants (Figs 1 vs. 2), but facilitation had a significant effect on *R:S* ratio. Facilitated plants had always smaller *R:S* ratio than isolated plants except in *Limonium* with *Stipa.* In three out of these 5 cases of facilitation, beneficiary plants had both greater root and shoot biomass than isolated plants.

**Figure 2:**
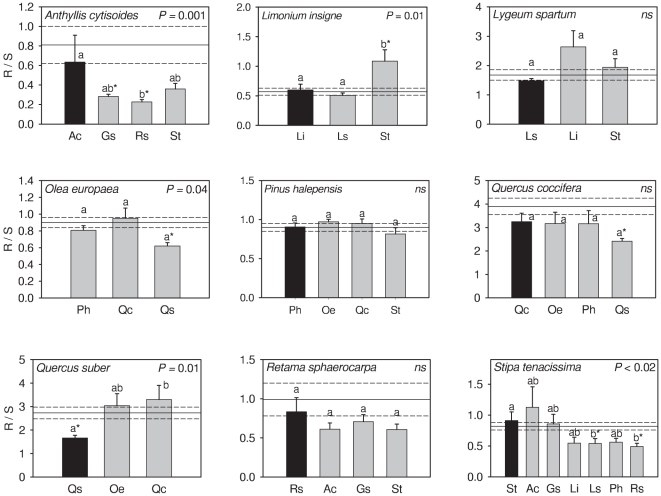
Root-to-shoot ratio of individuals of target species (name inside the panel) growing with individuals of the same (solid column) or different species (grey column). *P* is the significance of ANOVA (*ns*: non-significant differences). The continuous horizontal line shows mean values of R:S ratio for control plants and dashed lines represent ±1 SE. Bars with different letters are significantly different at *P*<0.05. Asterisk denotes significant differences with control plants. Legend of species is as in Table 1.

### Was there competition for light?

Although we assumed that plant competition for light was negligible in our experiment, we tested this assumption. Aboveground competition usually reduces *R:S* ratio due to an increase in shoot mass and a decrease in root mass [30]. We checked for differences in shoot mass and *R:S* ratios between control plants and individuals growing with a neighbour of similar or bigger size. There were no differences on shoot biomass among groups (*F*_1,456_  =  0.00, *P* = 0.99). By species, only shoot mass of *Anthyllis* and *Lygeum* growing with a conspecific differed from, and was smaller than, controls (3.99±0.43 *vs.* 2.30±0.61 g in *Anthyllis* and 0.96±0.10 *vs.* 0.64±0.05 g in *Lygeum*, for controls and pairs of conspecifics, respectively, *P*<0.05) but their *R:S* ratios were similar to that of isolated plants (Fig 2). Shoot mass of plants living with another species differed from controls only when facilitative effects were evident and never under competition; and when facilitation took place, *R:S* ratios were either smaller or similar to controls (Figs 1 *vs.* 2). These results suggest that either root facilitation counterbalanced shoot competition or that shoot mass of facilitated plants increased more than root mass. Overall, our analyses suggest that competition for light was negligible or in case it was present, its overall effect on plant performance was neutral.

### Effects on neighbours: Interaction intensity and competitive ability

**Figure 3:**
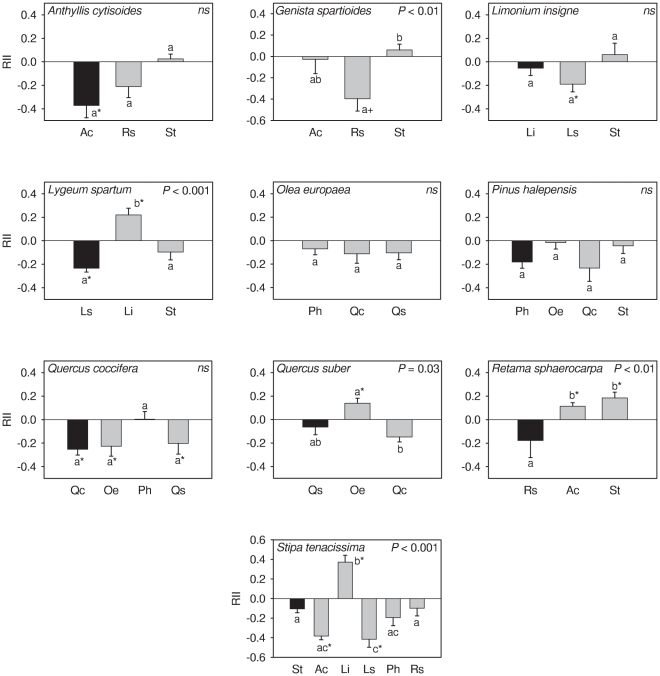
Net effect of target species on neighbour species. Intensity of the effects (*RII*) of species (name inside the panel) on individuals of the same (solid column) or different species (grey columns). *P* is the significance of ANOVA (*ns*: non-significant differences). Bars with different letters are significantly different at *P*<0.05. Asterisk and crosses denote, respectively, significant (* *P*<0.05) and marginally significant (^+^ *P*<0.07) differences with control plants, i.e. target species competed with or facilitated its neighbour. Legend of species is as in Table 1.

Seven out of 10 species analyzed did have a significant effect on the growth of at least one neighbour species (Fig 3). Intraspecific interactions had always negative or neutral net effects on plant performance, while interspecific interactions had either positive, neutral, or negative effects. The outcome and intensity of contest interactions was highly species-specific. Some species were very competitive, showing higher competitive abilities than all other species; for example *Quercus coccifera*, whose effect on other species was competitive except with *Pinus halepensis* whereas the effect of any other species on *Quercus coccifera* was neutral (Fig 3). There were, however, species that showed neutral, competitive, or facilitative effects on other species depending on the neighbour's identity, and that suffered competition or were facilitated by other species. For example, *Stipa* was a stronger competitor than *Anthyllis* (RII = −0.38±0.04 of *Stipa* on *Anthyllis* vs. 0.02±0.04 of *Anthyllis* on *Stipa*; *t* = 7.21, *P<*0.01) but both *Anthyllis* and *Stipa* had neutral effects on *Retama sphaerocarpa.* This later species, however, had a positive effect on both *Stipa* and *Anthyllis*. A similar case occurred with *Limonium*, *Lygeum* and *Stipa* (Fig 3).

## Discussion

Contest interactions had strong effects on plant growth even though all plants grew under homogeneous conditions with ample water and nutrient supply. Interactions were highly plastic, and while interactions with conspecifics had negative or neutral effects on growth, responses to heterospecific neighbours were highly variable, ranging from net positive to net negative effects. This specificity in the outcome resulted either in groups of species where a possible hierarchy in their competitive abilities could be hypothesised or groups where such rankings were not possible to establish.

By supplying enough resources above and below-ground we minimized the effects of resource depletion and maximized the effects unrelated to resource exploitation; i.e., contest competition or interference. This may be the main reason why, although generally negative, we found only 3 significant interactions among conspecifics (out of 8 cases) whereas intense intraspecific competition for resources is usually expected [4], [5]. However, heterospecific interactions were strongly asymmetrical and quite variable. Some species facilitated the performance of others (e.g., legumes on most other species, grasses on some small shrubs, and trees on other tree species) while other species always had negative (e.g., *Quercus* species) or neutral effects on neighbours. Our results also showed that one species may have different effects depending on its neighbour's identity, turning from being a strong competitor against some species to be facilitator for another species (e.g., *Stipa* or *Lygeum*). These results highlight the existence of strong specificity in interactions [31], [32] even under high resource supply. Overall, grasses, forbs, and small shrubs had more plastic responses to interactions than larger shrubs and trees, likely because of differences in growth rate and traits such as life span, morphology, or physiological traits allowing some species to respond faster to the presence of neighbours [33]–[35].

In the presence of neighbours, some species changed biomass allocation patterns which resulted in variations of *R:S* ratios. An increase in light competition usually results in increased allocation to shoots and decreased allocation to roots [30], while root competition usually increases, if anything, biomass allocation to roots [19], [20], [36]. Such responses, however, could result from growing in pots of different sizes, regardless of the presence of neighbours [6], [25], [30]. In our experiment soil volume was similar for plants growing alone or in pairs, and results showed that competitive interactions did not significantly change allocation patterns while, interestingly, facilitation decreased *R:S* ratios. Despite the importance of *R:S* ratio to plant performance, plasticity in *R:S* ratio is usually thought to be a poor predictor of competitive ability [20], [33], [37], and many reports showed no clear relationship between *R:S* and competitive ability [36]. However, none of such studies reported facilitative interactions. In our case, lower *R:S* ratios under positive root interactions did favour mass allocation to shoots–except for *Limonium* growing with *Stipa*–although it is worth noticing that all facilitated species had larger shoot and root biomass compared to controls–except for *Anthyllis* growing with *Retama*. Under favourable conditions roots can access plenty of resources and invest more in biomass aboveground and, ultimately, in enhanced reproduction [38].

We cannot distinguish whether facilitative belowground interactions enhanced plant performance due to niche complementary (different root depth or seasonality between interacting plants) or direct facilitation. All species were perennial, evergreen (except *Anthyllis*, a facultative summer-deciduous) and had similar growth seasonality. Pot size avoided big differences in spatial distribution of roots. Both facts suggest that niche complementary was not important in our experiment, and that the main process was probably due to direct root effects through exudates and release of secondary compounds. We found, for example, that legumes facilitated neighbour performance. Facilitation of legumes is often mediated by litter, but our experimental time span was too short to allow this process to be important. Recently, Ayres et al. [16] showed that nitrogen fixed by legumes can be directly transferred to other plants through root exudation or by shared mycorrhizae, and Li et al. [39] showed that legumes may have a direct facilitative effect on other plants by changing the pH or chemical composition of the soil *via* root exudates, mobilizing phosphorous which would otherwise be unavailable to the facilitated species. Roots are known to produce huge amounts of chemical compounds which are released in their surroundings; these exudates create the potential for highly species-specific contest interactions among plants and between plants and soil organisms [40], [41] which could lead to opportunities for non-hierarchical rankings of species competitive abilities in plant communities [6], [42].

Most research that explored interaction hierarchies in controlled environments found predictable competitive rankings if nutrients and water were sufficient [43], [44], supporting the idea that interactions are hierarchical and transitive. Although we did not address interactions among group of species, and thus we cannot clearly identify if there were or were not hierarchies in competitive abilities of target species, we can hypothesise some characteristics of such root interactions. We found species that acted as strong competitor with any other species (e.g., *Quercus coccifera*). However, we found species (e.g., *Stipa, Anthyllis* and *Retama*) with variable responses to root interactions. For them, rankings in competitive abilities were not evident, suggesting that root interactions might favour non-hierarchical networks when some species coexist. These results suggest intriguing questions that warrant to be tested: 1) the importance of contest vs. exploitation root interactions on the overall outcome of plant interactions; 2) whether the species-specific nature of root interactions found here can lead to species-specific outcomes of plant interactions in the field [31], [32]. Such cases may conflict with the paradigm of hierarchically based (transitive) community organization, and could support evidence for networks of interactions that may promote coexistence and community diversity through indirect effects [6], [42].

In summary, we found that plant responses to the presence of neighbours were variable. Plants in our experiment responded strictly to root-root interactions, conditioned by the type of neighbour and steered to contribute to competitive success, most likely codified by chemical signals not related to resources. Our results support the idea that roots can interact with their biotic and abiotic environments using a variety of mechanisms, far beyond the traditional view that plants interact mainly through resource depletion.
